# Social media in public health: an analysis of national health authorities and leading causes of death in Spanish-speaking Latin American and Caribbean countries

**DOI:** 10.1186/s12911-017-0411-y

**Published:** 2017-02-03

**Authors:** David Novillo-Ortiz, Tony Hernández-Pérez

**Affiliations:** 1 Office of Knowledge Management, Bioethics and Research, Pan American Health Organization (PAHO), 525 23rd ST NW, Washington, DC 20037 USA; 2 Department of Library Science and Documentation, University Carlos III de Madrid, Getafe, Spain

**Keywords:** Social media, Public health, eHealth, Latin America, Information retrieval

## Abstract

**Background:**

Information and communications technologies, like social media, have the potential to reduce some barriers in disease prevention and control in the Americas. National health authorities can use these technologies to provide access to reliable and quality health information. A study was conducted to analyze availability of information about the leading causes of death on social media channels of national health authorities in 18 Spanish-speaking Latin American and Caribbean countries.

**Methods:**

We gathered data of national health authorities’s institutional presence in social media. Exploratory-descriptive research was useful for analysis and interpretation of the data collected. An analysis was carried out for 6 months, from April 1 to September 30, 2015.

**Results:**

Sixteen of the 18 countries studied have institutional presences on social media. National health authorities have a presence in an average of almost three platforms (2.8%). An average of 1% of the populations with Internet access across the 18 countries in this study follows national health authorities on social media (approximately, an average of 0.3% of the total population of the countries under study). On average, information on 3.2 of the 10 leading causes of death was posted on the national health authorities’ Facebook pages, and information on 2.9 of the 10 leading causes of death was posted on their Twitter profiles. Additionally, regarding public health expenditures and the possibility of retrieving information on the leading causes of death, an apparent negative correlation exists in the case of Facebook, r(13) = −.54, *P* = .03 and a weak negative correlation in the case of Twitter, r(14) = −.26, *P* = .31, for the countries with presences in those networks.

**Conclusions:**

National health authorities can improve their role in participating in conversations on social media regarding the leading causes of death affecting their countries. Taking into account Internet accessibility levels in the countries under study and the high rates of people using social networks in even the poorest countries, further research is needed to provide evidence that more dedication to health promotion interventions through social media could significantly improve the impact and reach of public health messages and initiatives.

**Electronic supplementary material:**

The online version of this article (doi:10.1186/s12911-017-0411-y) contains supplementary material, which is available to authorized users.

## Background

Public health in the Americas has experienced significant improvements over the last few decades. However, considerable challenges persist in the prevention and control of diseases. Suboptimal levels of maternal and child health, insufficient human and infrastructure resources, and wide geographical and cultural differences add further complexity to the situation in the region [1]. There are 23 leading causes of death in countries of the Americas, some of which are preventable. Three examples illustrate the preventable nature of major causes of death: diabetes can be largely prevented by a healthy diet and lifestyle; HIV/AIDS can be prevented by taking adequate precautions during sex; and finally, interpersonal violence is a scourge that must be confronted by the integration of actions in many sectors of society [2]. In all of these cases and in many others, access to reliable and quality health information and appropriate medical advice can contribute to a dramatic reduction in the mortality figures of these countries. From the Declaration of Alma-Ata [3] to the Millennium Declaration [4], access to reliable health information and knowledge sharing, through use of information and communication technologies, has been considered essential for health development [5].

In a setting where a large amount of content is available about different health topics, some of which is produced by institutions with conflicting interests (i.e., the pharmaceutical industry), and which can be consumed through different channels (e.g., mass media, search engines, social networks, etc.), governments must be the preferred source of information for citizens to consult for health information. One of the key tools governments have available to provide access to information is their presence on the Internet, particularly through their social media profiles. In order to address these realities, we conducted a study to analyze the presence of national health authorities on social media in 18 Spanish-speaking Latin American and Caribbean countries and the availability of information on the leading causes of death on the social media profiles of national health authorities. This study refers to the actions that national health authorities can take on the Internet (regarding the use of social networks) to share and make available information of interest on their 10 leading causes of death. The information gathered intends to motivate national health authorities to reach out to their populations with Internet access by increasing their activity on social networks.

## Methods

**Fig. 1:**
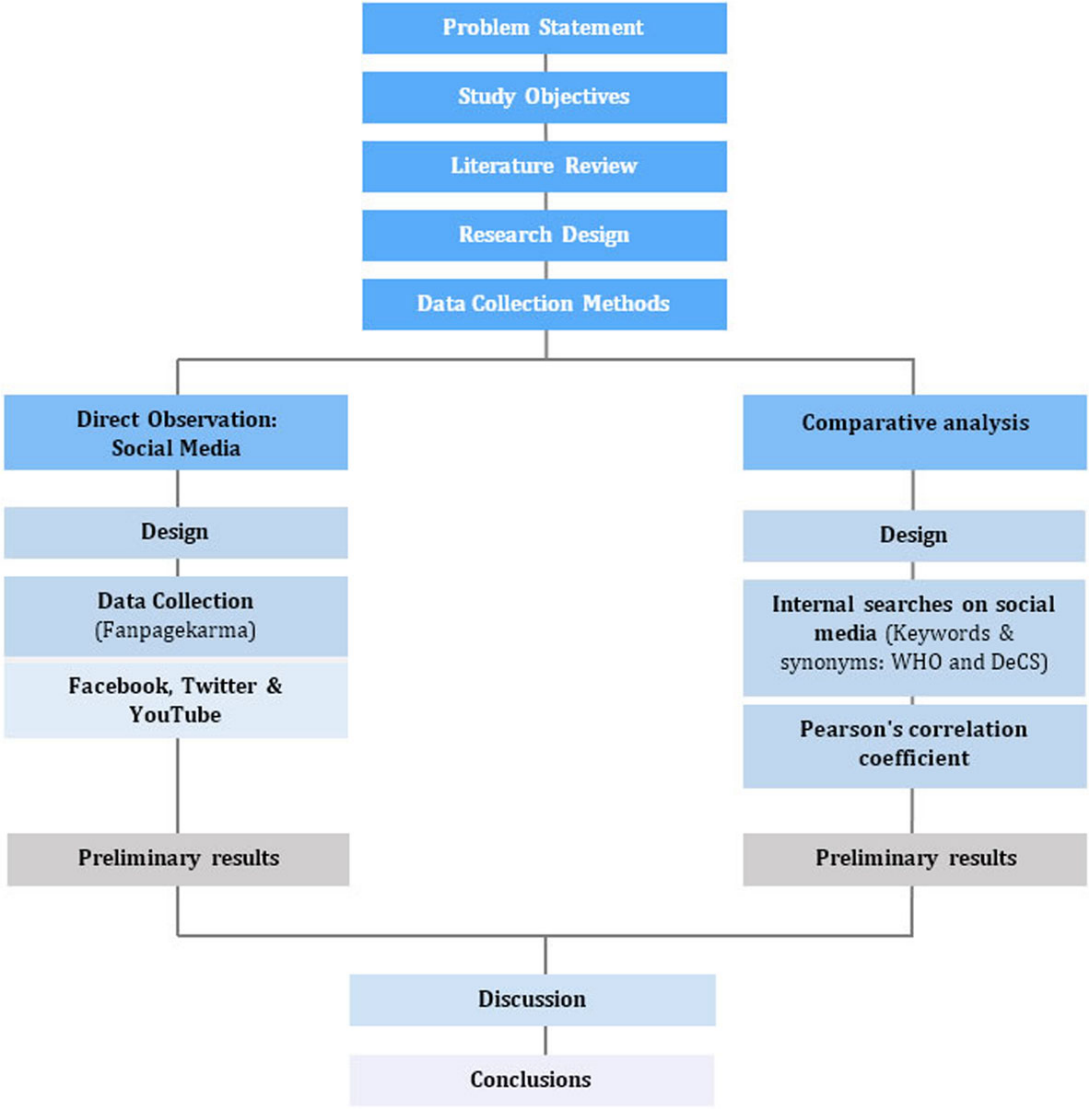
Research design

The research methods selected to undertake this research were the following: a literature review for the development of the conceptual framework, data collection by structured and direct observation about social media presence of national health authorities, and a search for information about the leading causes of death on their online institutional profiles. A comparative analysis by country was conducted on the collected data (Fig. 1).

### Target population

**Fig. 2:**
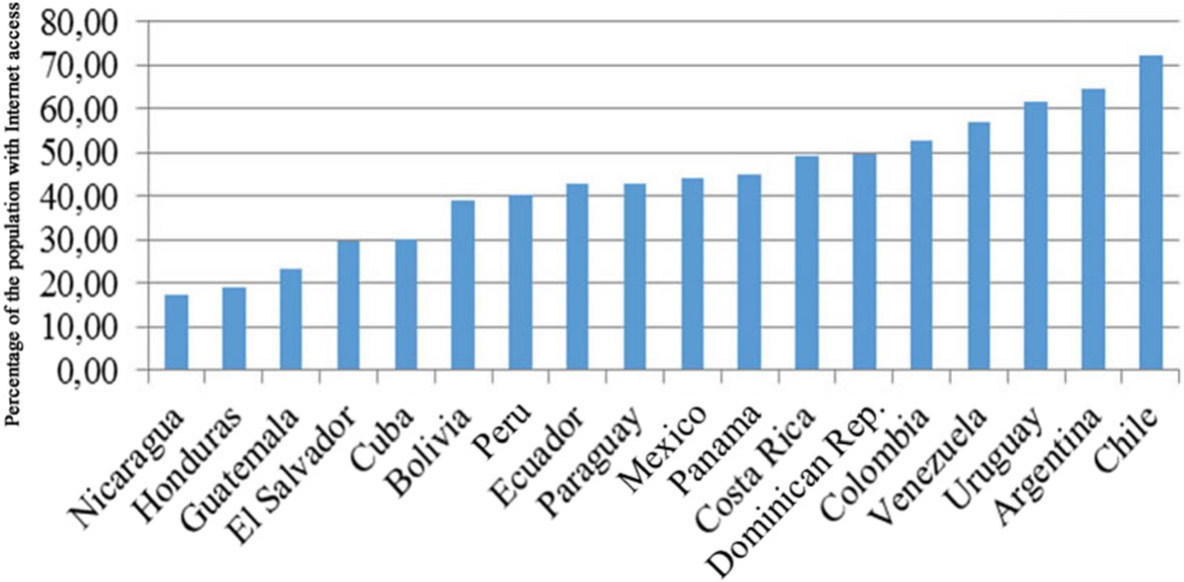
Percentage of the population with Internet access in Spanish-speaking Latin American and Caribbean countries

The Americas is the region with the second-highest Internet access rates, 66% compared to 77.6% in Europe [6]. In Latin America, specifically in the 18 countries part of this study, a total of 188 million people are connected to the Internet. The percentages of penetration vary significantly when considering that, for instance, in Chile Internet penetration reaches 72%, compared to Nicaragua where the proportion of the population with Internet access is 17%. Considering total connectivity data, the median penetration in the analyzed countries is 44.6% (Fig. 2).

Regarding social media penetration, in 2013, 78.4% of Internet users in Latin America participated in social networks—a percentage significantly higher than that of North America (64.6%) and Western Europe (54.5%)—even though the latter two had higher Internet penetrations than Latin America [7]. Additionally, Latin American users spend the most time using social media, with an average of 8.6 h/month per visitor. Regarding audience profile, no gender differences were found in any region, with 50% women and men visiting social media sites; the main audience (60.5%) consists of people 15–34 years of age. With regards to the most visited social media sites, Facebook is the market leader with 95.6% of time consumed on social media [8].

The national health authorities in 18 Spanish-speaking countries in Latin American and the Caribbean are the focus of this study. These countries are: Argentina, Bolivia, Chile, Colombia, Costa Rica, Cuba, Dominican Republic, Ecuador, El Salvador, Guatemala, Honduras, Mexico, Nicaragua, Panama, Paraguay, Peru, Uruguay, and Venezuela. The variables surveyed were the presence of these institutions in social media (Facebook, Twitter, and YouTube). Sixteen profiles on Twitter, 15 profiles on Facebook, and 12 profiles on YouTube were analyzed for a period of 6 months (from April 1 to September 30, 2015). Within the framework of this work, “national health authorities” means those institutions that, by constitutional mandate, are the public health governing bodies of a country, namely, health ministries and secretariats at the national level.

### Selection of terminology

**Fig. 3:**
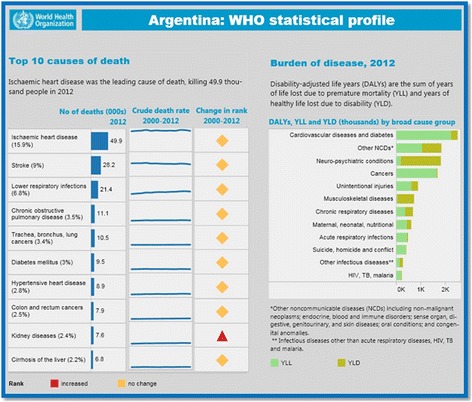
Sample country (Argentina) health profile issued by WHO, 2012

Official WHO country health profiles [9] were consulted to find the 10 main causes of death, which served as reference for the selection of terminology. Figure 3 shows a sample country health profile issued by WHO. It is worth mentioning that this information was last updated by WHO in 2012. Synonyms were also used to broaden the possibilities of retrieving information in the platforms searched. The DeCS (Descriptors in Health Sciences) controlled vocabulary [10] was used to facilitate the search for synonyms. In addition, Google Trends was used to detect other synonyms for the terms used to trace information on top causes of death [11]. It was clear from the search that there were no synonyms different enough to affect the total number of results retrieved. The list of keywords and synonyms used (in Spanish) on leading causes of death is included in Additional file 1.

According to the analysis of the 18 countries under study, 23 leading causes of death affecting these countries were identified. Since the official information on leading causes of death was originally provided in English, the Pan American Health Organization (PAHO, Regional Office of WHO in the Americas) website [12] was used for translation into Spanish and to ensure the consistency of Spanish terminology used in Latin American countries (see Table 1).

**Table 1:** Leading causes of death keywords for the countries under study

Stroke (*accidente cerebrovascular*)	Hypertensive heart disease (*enfermedad cardiaca hipertensiva*)
Congenital anomalies (*anomalías congénitas*)	Alzheimer’s disease (*enfermedad de alzhéimer*)
Birth trauma and asphyxia (*asfixia y trauma en el nacimiento*)	Kidney disease (*enfermedad renal*)
Colorectal cancer (*cáncer de colon y recto*)	Diarrheal disease (*enfermedades diarreicas*)
Stomach cancer (*cáncer de estómago*)	Acute lower respiratory infection (*infección aguda de las vías respiratorias inferiores*)
Breast cancer (*cáncer de mama*)	Traffic accidents (*lesiones en carretera*)
Prostate cancer (*cáncer de próstata*)	Protein-energy malnutrition (malnu*trición proteico-energética*)
Lung cancer (*cáncer de pulmón*)	Chronic obstructive pulmonary disease (*neumopatía obstructiva crónica*)
Ischemic heart disease (*cardiopatía isquémica*)	Tuberculosis (*tuberculosis*)
Liver cirrhosis (*cirrosis hepática*)	HIV/AIDS (*VIH/Sida*)
Complications from premature birth (*complicaciones del parto prematuro*)	Interpersonal violence (*violencia interpersonal*)
Diabetes (*diabetes*)	

### Exploratory and descriptive analysis

For the exploratory and descriptive analysis of the presence of national health authorities on social networks, Facebook, Twitter, and YouTube were used as key sources of information. Only institutional profiles that were accessible from health authorities’ official websites were used as sources. The presence of national health authorities on Google+, Instagram, and Flickr was negligible, so an examination of these platforms was not undertaken.

**Fig. 4:**
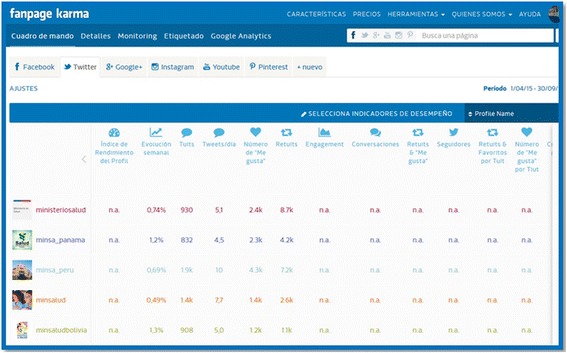
Fanpagekarma Interface

In order to capture data and analyze the presence of national health authorities on social media, the Fanpagekarma tool was used (see Fig. 4) [13]. Fanpagekarma makes searching content easier--by downloading data in Excel format for Facebook, and by a direct search on the platform for Twitter)—using the selected terminology (keywords and selected synonyms). This tool made it possible to obtain a large number of analysis indicators for the three platforms surveyed. On Facebook, the following indicators were analyzed, among others: number of followers, mean posts per day, “Shares” per post, “Comments” per post, and “Likes” per post. On Twitter, the analyzed indicators were number of tweets, tweets per day, favorites, and retweets. The analyzed indicators of YouTube were total number of videos and total number of views.

### Comparative analysis

After mapping the institutional presence on social media, an analysis was conducted on whether national health authorities include information on the leading causes of death on their social media profiles, mainly Facebook and Twitter. A correlation, indicated by Pearson’s correlation coefficient, *r*, measures the strength and the direction of a linear relationship between two variables: the public health expenditure and the number of leading causes of death found on Facebook and Twitter. According to the World Bank, “public health expenditure (% of Gross domestic product -GDP-)” consists of recurrent and capital spending from government (central and local) budgets, external borrowings and grants (including donations from international agencies and nongovernmental organizations), and social (or compulsory) health insurance funds [14].

### Limitations

The study and the analysis were conducted in Spanish. The purpose of this restriction was to ensure the consistency of results and facilitate the comparison of results obtained in the countries involved. Special mention should be made of the exclusion of Brazil, a Portuguese-speaking country and the largest Latin American country. It was excluded only because of the language criteria that ensured the consistency of the terminology used. Nevertheless, the recommendations included in this study can guide any country willing to work on strategies to provide access to health-related information.

Additionally, the target audience to whom specific health interventions are directed through the use of social networks is exclusive to persons with Internet access. It should be remembered that Internet access is still limited in the countries surveyed and that Internet access is more readily available to the more affluent who frequently also have better access to healthcare. Therefore, other actions on health promotion not related to social networks are beyond the scope of this study.

### Literature review

At least six countries in Latin America have conducted studies on the use of social networks in the context of health (Argentina, Brazil, Colombia, Guatemala, Mexico, and Peru). The recurrent topics in these studies are HIV/AIDS and tobacco. Regarding HIV, the use of social networks was found useful in encouraging discussions around HIV testing and in addressing AIDS-related stigma [15, 16]. With regard to tobacco, the studies reveal that proposed legislation to ban tobacco advertising needs to include Internet sites and related social media [17]. Moreover, an institution in Mexico conducted a study that demonstrated the apparent interest of the non-professional population to follow social network initiatives about health communications and the diffusion of material on the fight against tobacco use. Nevertheless, more research is necessary in order to recognize the real impact of changing behavior [18].

Other prior studies on the use of social media by health authorities examined patterns and characteristics of social media adoption in state health departments in the United States in 2012. The presence of health departments on social media increased from 28 in 2008 to 41 in February 2012. The trends analyzed showed that the adoption of social media by state departments of health was associated with residential areas where Internet penetration levels were higher. It was determined that the presence on social media was positively associated with the development and dissemination of communication strategies on public health [19]. Another study found that the presence on social media recalls the trend towards unidirectional communication when the web came about, when the core idea was to have a presence, beyond the logic of why being present and of how to manage all the matters related to an institutional image [20].

One additional study showed how health institutions used Facebook, Twitter, and YouTube mostly to disseminate information about the organization, followed by information about health education [21]. In another study it was concluded that health authorities should adopt and apply this type of technology to assess, protect, and promote public health [22].

## Results

### Presence of national health authorities on social media

The analysis of the presences of national health authorities showed that these institutions have presences on six different social media, namely: Facebook, Twitter, YouTube, Flickr, Google+, and Instagram. Sixteen of the 18 countries studied (*N* = 18) have an institutional presence on at least one of the six social media mentioned. Specifically, almost 90% (88.8%) of the sample countries have an institutional presence on Twitter and Facebook with 16 and 15 out of 18, respectively. YouTube, with 61.1% (11 out of 18), is the third platform with a significant institutional presence of national health authorities. Proportions are lower on other platforms: Flickr with 22.2% (4 out of 18), Google + with 16.6% (3 out of 18), and Instagram with 5.5% (1 out of 18). The information about the presence of national health authorities on social media is included in Additional file 2.

National health authorities have presences on an average of almost three social media platforms. Only Cuba and Nicaragua have no institutional presence on any of the social media mentioned. Keeping in mind that the social media with the largest institutional presences are Facebook, Twitter, and YouTube, these three platforms were used as sources for the analysis of activity and information retrieval. All the information relevant to the analysis of these three platforms is available in the Additional file 3.

### Facebook

Fifteen out of the 18 countries under study have institutional presences through their national health authorities on Facebook (Argentina, Bolivia, Chile, Colombia, Costa Rica, Dominican Republic, Ecuador, El Salvador, Guatemala, Honduras, Mexico, Panama, Paraguay, Peru, and Uruguay). Costa Rica, Panama, and Peru are the countries with the highest proportion of Internet users who follow national health authority profiles on Facebook (2.99%, 1.43%, and 1.10%, respectively – in turn representing 1.47%, 0.64% and 0.44% of the total population of those countries-, respectively). Uruguay (0.13%), Honduras (0.13%), and Colombia (0.14%) are the countries with the fewest followers in relation to the population with Internet access, representing 0.08%, 0.02% and 0.07% of the total population of those countries, respectively. The information about the population with Internet access by country following national health authorities on Facebook is included in Additional file 4.

The mean number of Facebook posts is 3.8 per day. Peru’s national health authority has the largest number of posts per day, with almost 13 (12.9 posts/day) compared to Costa Rica, which posts with a lower frequency (0.1 posts/day). For each post, the favorite option of followers is to click the “Like” button (including all the countries under study, the average of “Likes” per post is 90), followed by the possibility of sharing in their network (each individual post, considering the mean of all the countries, is shared 54 times, on average). The least-used option is to comment on posts (each individual post receives a mean of 3.6 comments per post).

Information on leading causes of death was searched in the text of posts during the period under study. This exercise was to identify the most common interests of users and to determine whether national health authorities posted contents related to leading causes of death in their countries. Information on the ten leading causes of death by country was also searched on each Facebook profile, including keywords and synonyms. On average, information on three of the ten leading causes of death was posted on the national health authority’s Facebook site.

### Twitter

Sixteen out of the 18 countries under study have institutional presences through their national health authorities on Twitter (Argentina, Bolivia, Chile, Colombia, Costa Rica, Dominican Republic, Ecuador, El Salvador, Guatemala, Honduras, Mexico, Panama, Paraguay, Peru, Uruguay, and Venezuela). The countries with the highest proportion of Internet users who follow national health authority profiles on Twitter are Ecuador (2.37%), El Salvador (1.88%), and Peru (1.64%), representing 1.02, 0.55 and 0.66% of the total population of those countries, respectively. Uruguay (0.12%), Guatemala (0.11%) and Bolivia (0.12%) are the countries with the smallest number of followers in relation to the population with Internet access, representing 0.06, 0.02 and 0.04% of the total population of those countries, respectively. The information about the population with Internet access following national health authorities by country on Twitter is included in Additional file 4.

The average number of tweets per day is 7.1. The national health authority with the largest number of posts per day is Venezuela, with a mean of 19.1 tweets/day compared to Costa Rica, which posts 0.1 tweets/day. For each message sent through Twitter, the preferred option of users is to retweet a message rather than mark it as a favorite. Specifically, the average of favorites per tweet is 2.6 and the mean for retweets per tweet sent is 3.3. The average number of all messages retweeted and marked as favorite per tweet is 5.9.

Information posted by national health authorities on leading causes of death was searched on Twitter with the same methodology used for Facebook. Each Twitter profile was searched for the 10 leading causes of death by country, including keywords and synonyms. On average, users were able to access information on 2.9 of the 10 leading causes of death posted on the national health authority’s Twitter profiles.

### YouTube

Twelve of the 18 (66.6%) national health authorities of the countries analyzed have a presence on YouTube (Argentina, Bolivia, Chile, Colombia, Costa Rica, Dominican Republic, Ecuador, El Salvador, Mexico, Panama, Paraguay, and Peru). The average number of videos analyzed per channel was 313. The average number of views per profile was 1 million, and the average number of views per video was 4500. Analyzing the content of the most viewed videos for the period studied, 3 of the 12 YouTube profiles analyzed (Argentina, Chile and Paraguay) shared videos addressing some of the 10 leading causes of death affecting their countries.

### Socio-economic status and social media

Internet accessibility rates are lower in poorer countries, concentrated mostly in sub-Saharan Africa and parts of Asia, including 39% in Nigeria, 30% in Indonesia and 22% in India. In Latin America, a median of 64% of the population has access to the Internet, with the highest rates in Chile (78%) and Argentina (71%) [23]. Specifically, for the 18 countries targeted in this study, the median Internet penetration is 44.6% [6]. One study suggested that individuals with different levels of socioeconomic status vary in the heuristics and search patterns they rely upon to direct their searches. The researchers found that people with limited resources may be disadvantaged when turning to the Internet to make a health decision [24]. In our research, socio-economic variables were also considered to identify whether the socioeconomic status of different countries correlates with reach in social media platforms. Countries like Mexico, Argentina, Peru, Chile, and Colombia are among the ten countries with the widest reach in social media sites, thus leading to the conclusion that no correlation exists between social media reach and income levels [7].

**Fig. 5:**
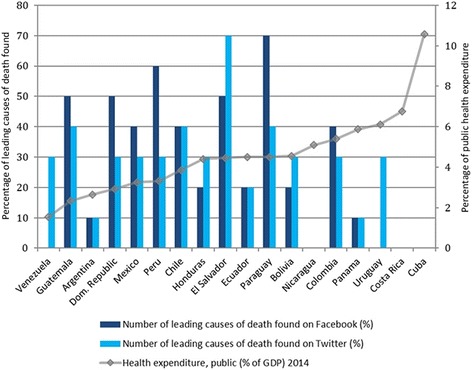
Public Health Expenditure and Number of leading causes of death found on Facebook and Twitter, by country, 2015

Another variable considered was whether a correlation exists between public health expenditure and the possibility of retrieving information on the leading causes of death through social media (see Fig. 5). The aim was to show whether public health expenditure is a limitation to having good visibility in each country’s content on social media. Seven of the 11 countries whose public health expenditures are below the mean (Guatemala, Dominican Republic, Mexico, Peru, Chile, El Salvador, and Paraguay), exceed average information retrieval on the 10 leading causes of death on Facebook and Twitter. On the other hand, 6 out of the 7 countries that surpass average public health expenditures (Bolivia, Costa Rica, Panama and Uruguay; together with Cuba and Nicaragua, with no presence on Facebook and Twitter), do not reach the average of information retrieval on their 10 leading causes of death in Facebook.

After calculating the Pearson correlation coefficient, the result shows an inverse correlation for Facebook and Twitter, vis-à-vis public health expenditure (as a percentage of GDP) and the possibility of finding information on the leading causes of death. For a country with a presence in both aforementioned networks, the correlation is negative: in the case of Facebook it is r(13) = −.54, P = .03 and for Twitter it is r(14) = −.26, P = .31 (not as strong as Facebook, yet negative nonetheless).

## Discussion

### Internet penetration

There is a digital gap in Internet penetration in Latin America, particularly in the countries under study, where we can find countries with an Internet penetration higher than 70% (Chile) and others with a penetration index that does not reach 20% (Nicaragua), and where median penetration is 44.6%. In only five of the 18 countries – accounting for 27.7% of the total – the penetration rate is higher than 50%. These countries are Colombia, Venezuela, Uruguay, Argentina, and Chile. Poor technological infrastructure, particularly regarding Internet access, may represent a regression concerning the use of social networks in the field of public health. Specifically, an average of only 1% of the population with Internet access across the 18 countries in this study follows national health authorities on social media, which represents approximately 0.3% of the total population of the countries under study.

Social media are widely used to search for health-related information. They provide national health authorities with unique opportunities to satisfy the needs of the population to access health information by providing education and information to their followers. In addition, the sum of interactions with health institutions on social media can help the population seeking health information on the Internet by offering reliable information in contrast to sites of doubtful accuracy [25, 26].

Fifteen out of the 18 countries under study have institutional presence on Facebook through their national health authorities, when the activity rate was below the mean (1.6), the main reason was that the number of posts on those platforms was well below the average (692 posts) (see Table 2). A priori, it appears that there is no relationship between income levels and reach in social media sites. Nevertheless, six out of the seven countries that surpass average public health expenditure do not reach the average of information retrieval on their 10 leading causes of death on Facebook.

**Table 2:** Activity rate for Facebook followers of national health authorities

Country	N° of publications	Population with Internet access		Users who follow national health authority		Activity rate (“Like,” “Comment,” “Share”)	
		Absolute value	Percentage	Absolute value	Percentage of people with Internet access	Absolute value	Percentage
Costa Rica	17	2,350,733	49.41	70,355	2.99	2,065	0.02
Panama	112	1,737,297	44.92	24,858	1.43	2,494	0.10
Dominican Rep.	370	5,159,267	49.58	11,477	0.22	5,053	0.44
Ecuador	240	6,838,254	43.00	72,975	1.06	34,828	0.47
El Salvador	305	1,813,989	29.70	11,481	0.63	9,964	0.86
Argentina	326	27,808,077	64.70	221,511	0.79	287,722	1.29
Guatemala	292	3,747,626	23.40	6,016	0.16	8,243	1.37
Uruguay	78	2,101,635	61.46	2,822	0.13	4,554	1.61
Bolivia	956	4,121,248	39.02	7,400	0.17	12	1.62
Colombia	296	25,123,935	52.57	36,257	0.14	62,002	1.71
Chile	1,411	12,851,275	72.35	71,073	0.55	148,269	2.08
Paraguay	1,869	2,817,583	43.00	22,823	0.81	51,275	2.24
Mexico	1,229	55,658,771	44.39	158,021	0.28	432,394	2.73
Peru	2,375	12,451,205	40.20	137,313	1.10	431,425	3.14
Honduras	505	1,519,089	19.08	1,960	0.13	10,477	5.34
Mean values	692					100,000	1.66

### Population with internet access following national health authorities on facebook and twitter

**Fig. 6:**
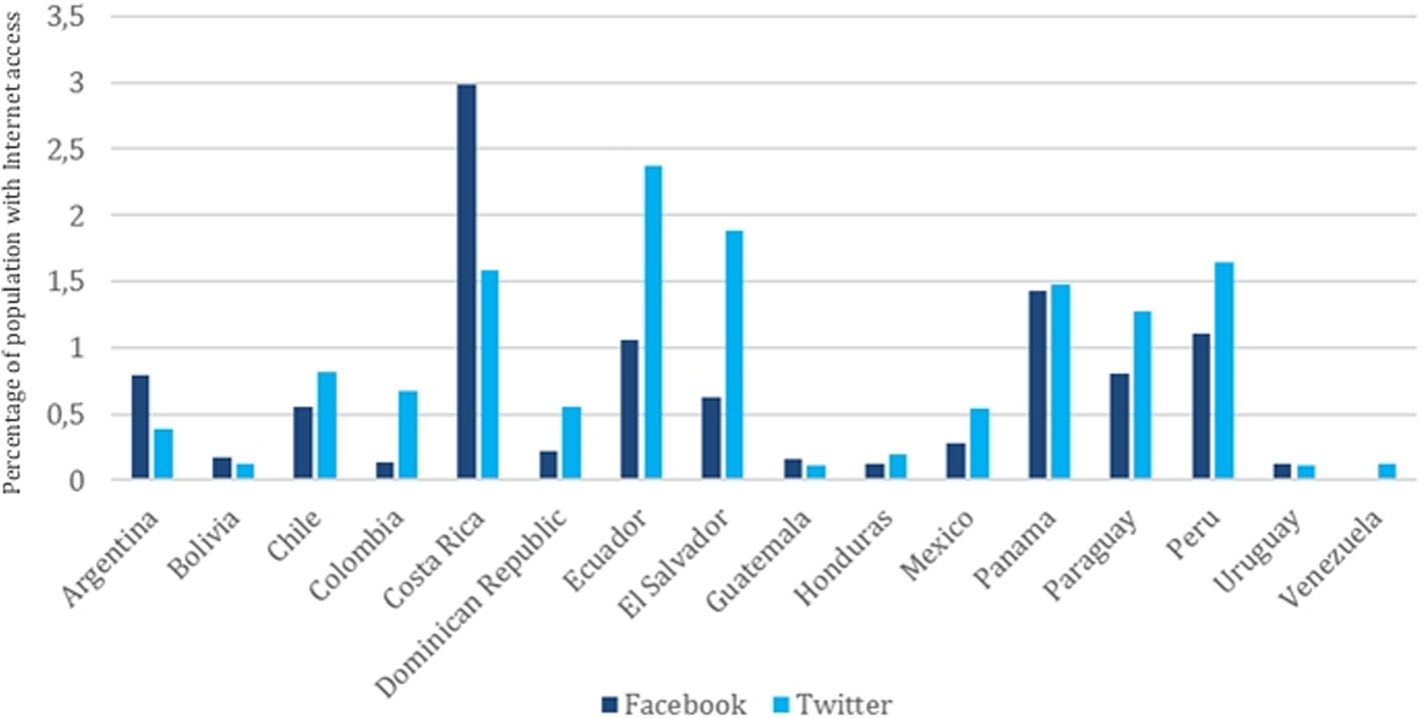
Percentage of population with Internet access following national health authorities on Facebook and Twitter

Based on the assumption that followers of national health authorities’ profiles on social media were born and/or live in those countries, on average 1% of the population with Internet access, across the 18 countries in this study, follows national health authorities on social media (see Fig. 6), which represents approximately 0.3% of the total population of the countries under study. Even though Facebook is the most widely used social network in the Americas [7], for the objectives of this study Twitter is the social network in which the national health authorities have the most followers. On Twitter, the mean number of the total population with Internet access following national health authorities is 0.86% - representing 0.33% of the total population of the countries under study-, with Ecuador (2,37%), El Salvador (1,88%), and Peru (1,64%) being the three countries with the highest number of followers compared to the total population with Internet access, representing 1.02, 0.55 and 0.66% of the total population of each country, respectively. In the case of Facebook, the mean number of the population with Internet access following national health authorities is 0.79% which represents approximately 0.28% of the total population of the countries in this study. Costa Rica (2.99%), Panama (1.43%) and Peru (1.10%) are the countries whose national health authorities have the most followers, representing 1.47, 0.64 and 0.44% of the total population of each country, respectively.

This study provides a valuable perspective on which countries have the best opportunity to influence their communities based on the content shared. In the six countries where at least 0.8% of the population with Internet access follows national health authorities on Facebook, information on one or none of the leading causes of death can be retrieved in four countries, namely, Costa Rica (2.99% of the population with Internet access, 1.47% of the total population), Panama (1.43% of the population with Internet access, 0.64% of the total population), Ecuador (1.06% of the population with Internet access, 0.44% of the total population), and Argentina (0.8% of the population with Internet access, 0.51% of the total population). In the case of Twitter, the countries in the same situation are Ecuador (2.37% of the population with Internet access, 1.02% of the total population), Costa Rica (1.59% of the population with Internet access, 0.78% of the total population), and Panama (1.48% of the population with Internet access, 0.66% of the total population).. Although it is possible that health authorities in these countries can reach the population with their messages, it seems that they are missing the opportunity to share country-specific information on leading causes of death with a large audience. The information about the population with Internet access following national health authorities by country on Facebook and Twitter is included in Additional file 4.

The number of messages posted on Twitter by national health authorities is almost twice that of messages posted on Facebook. An average of 7.1 tweets is posted daily compared to 3.7 posts on Facebook. Nevertheless, the number of interactions per post elicited by these messages was 147.6 on Facebook versus 5.9 on Twitter.

### Activity rate of users and information retrieval on facebook and twitter

Another variable to consider regarding the number of interactions is the activity rate of users, defined as the total number of interactions divided by the total number of followers. On Facebook these interactions are “Like,” “Share,” and “Comment,” and on Twitter, “Favorites” and “Retweets.” This rate can identify activity as well as the strength of the relationship between followers and national health authorities. It also helps to define the countries in which national health authorities can have a greater influence on health decision-making due to the relationships existing between users and authorities. A higher activity rate implies an increased possibility to influence the public health of an active community interested in interacting with the contents produced by national health authorities. A comparison of activity rate and information retrieval on Facebook and Twitter is included in Additional file 5.

On Facebook, the countries with the highest user activity rates are Honduras (5.3), Peru (3.1), and Mexico (2.7). The countries with the highest user to national health authority rates on Twitter are Honduras (5.8), Uruguay (1.3), and Venezuela (0.8). When the activity rate on Facebook was below the mean (1.6), it was observed that the main reason for the low activity rate was that those profiles had posted well below the average, which was 692 posts per profile. It was not possible to identify such a pattern with Twitter. This may be due to the different ways in which these social media work. As previously observed, there is no doubt that the impact of a post on Facebook is higher than on Twitter and that users spend less time on Twitter than on Facebook, and it follows that the possibility of interaction between users and national health authorities is also lower. A tweet can rapidly fade out based on the time users are connected to Twitter and on the number of people followed by users, thus making it difficult to identify potentially relevant messages or those of interest for users. Still, more research is needed to interpret the greatest number of interactions on Facebook versus Twitter beyond the possible relationship between a greater number of interactions when there are more publications.

**Fig. 7:**
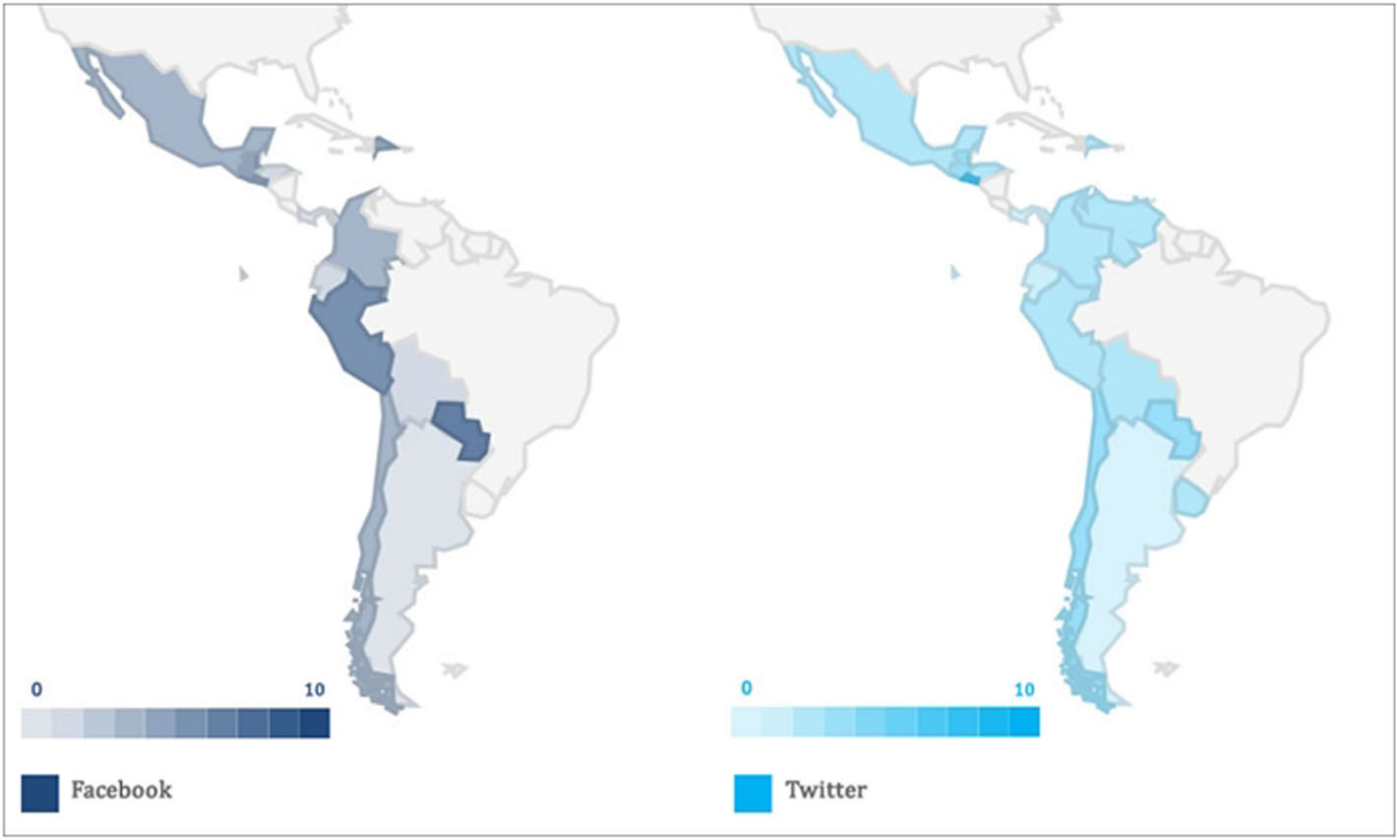
Number of leading causes of death found on Facebook and Twitter, by country

Taking into account the analysis of information retrieval on causes of death posted by national health authorities in their institutional profiles on Facebook and Twitter (during the period April–September, 2015) and the analysis conducted about activity rate on social media (see Fig. 7), two of the three countries exceeding average information retrieval on causes of death on Facebook, Paraguay and Colombia, have, in turn, an activity rate (2.2 and 1.7 on social media, respectively) exceeding the average for the countries under study (1.6). This means that the followers of these profiles on Facebook have a higher chance of accessing contents on the leading causes of death in their countries. The cases of Peru, Mexico, and Chile, having activity rates of 3.1, 2.7, and 2.1, respectively, are similar. If the activity rates of these countries on social media are considered, and assuming that followers are involved with health-related issues, there is a higher chance of promoting a particular behavior or call to action on a particular issue. On the other hand, it is worth mentioning the case of Honduras, which has the highest activity rate (5.3) but is currently among the countries with retrieval of information for one or none of the leading causes of death. Since it was not possible to retrieve information with the selected search strategies for Honduras, sharing information on causes of death could have a greater beneficial impact on the community compared to other countries where community activity is lower. Therefore, the capacity to impact the behavior of the population is also lower.

Something similar to Facebook occurs on Twitter. Honduras and Venezuela, two of the three countries with an activity rate higher than the average (5.8 and 0.8, respectively), only offer information on three of the 10 leading causes of death on Twitter. Attention should be given to the possibilities of both countries positively influencing their Twitter community by making information on leading causes of death in their countries available.

### Socio-economic impact

Aspects such as cost reduction and high internet penetration might explain why countries such as Mexico, Argentina, Peru, Chile and Colombia are among the 10 countries with the widest reach in social media platforms [23]. The existence of an apparent negative correlation between public health expenditure and the number of the leading causes of death that can be found on Facebook (−0.54) could suggest that for those countries in which the average public health expenditure is not exceeded, social media platforms could be considered low-cost tools used to reach the public. Further research is needed to provide evidence that more dedication to health promotion interventions through social media could significantly improve the impact and the reach of public health messages and initiatives on the population.

## Conclusions

The large number of institutional profiles on social media shows that national health authorities are aware of the relevance of having a presence on social media. Nevertheless, national health authorities can still improve in terms of the role they can play and their participation in conversations on social media regarding the leading causes of death that affect their countries. The analysis conducted shows that Facebook can be a useful tool for health promotion interventions. Taking into account the penetration level of the Internet in the countries under study, more dedication to public health expenditures—mainly in those countries with public health expenditures above average, but with a low possibility of retrieving information on leading causes of death—for health promotion interventions through social media could significantly improve the impact and the reach of public health messages. Further research is needed to know the exact budget allocated to this type of intervention as well as to identify and compare penetration to other traditional interventions, such as press, radio and/or television.

Finally, some recommendations for policy makers based on this paper are:

- Along with national statistics agencies, perform an exhaustive analysis of the population connected to the Internet, mainly considering the variables of age and sex, including questions about the use of social media or queries about health on the Internet. This will allow better planning of measures regarding health messaging, including the composition of messages with proper language and aimed at the appropriate audience.
- Based on the data obtained, appraise the use of the social network Twitter as one of the main communication platforms in emergency and disaster situations, including everything related to health alerts. Moreover, consider the social network Facebook as one of the main platforms to reach out to the population for community building and development, mainly at the time of raising awareness of the population in terms of public health campaigns (obesity, interpersonal violence, diabetes, etc.).
- Promoting and participating in conversations on social media may help to improve institutional image and to discover what people think of the services offered by health institutions. Non-traditional communication channels can help to improve the quality of information that can be shared and can keep that information up to date.
- We suggest reviewing the vocabulary used when referring to public-health-related terms, particularly to ensure overall consistency (i.e., that the terms used are the same every time a message is disseminated), as well as to facilitate information retrieval. This review should consider whether the terminology used is the same the public would use, taking into account that it would be citizens who will finally need to consult and receive advice on the content.

## Additional files

Additional file 1: List of keywords and synonyms on leading causes of death used (Spanish). (PDF 136 kb)
Additional file 2: Summary table of information about the presence of national health authorities on social media. (PDF 220 kb)
Additional file 3: Summary table of the analysis of the use of Facebook, Twitter and YouTube by national health authorities. (PDF 392 kb)
Additional file 4: Summary table of information about the population with Internet access following national health authorities by country on Facebook, Twitter and YouTube. (PDF 132 kb)
Additional file 5: Summary table showing a comparison of activity rate and information retrieval on Facebook and Twitter is included. (PDF 321 kb)

## Abbreviations

DeCS: Descriptors in health sciences
GDP: Gross domestic product
MeSH: Medical subject headings
NLM: National library of medicine
PAHO: Pan American health organization
WHO: World Health Organization
