# Supplementary material for: Social media in public health: an analysis of national health authorities and leading causes of death in Spanish-speaking Latin American and Caribbean countries
Source: BMC Med Inform Decis Mak. 2017 Feb 3;17:16. doi: 10.1186/s12911-017-0411-y (PMC5291998; doi:10.1186/s12911-017-0411-y)
Supplement: Additional file 1: — List of keywords and synonyms on leading causes of death used (Spanish). (PDF 136 kb) [file 12911_2017_411_MOESM1_ESM.pdf]

Additional file 1. List of keywords and synonyms on leading causes of death. Source: DeCS (Spanish)

Causa de defunción (palabra clave utilizada por OMS)	Sinónimos (DeCS)
**Accidente cerebrovascular**	
	Ataque
	Ictus
	Ictus Cerebral
	AVC
	Apoplejía
	Ataque Cerebral
	Apoplejía Cerebral
	Apoplejía Cerebrovascular
	Derrame Cerebral
	Accidente Vascular Encefálico
	AVE
	Accidente Vascular del Cerebro
	Accidente Vascular Cerebral
	Accidente Cerebral Vascular
	Accidentes Cerebrovasculares
**Anomalías congénitas**	
	Anomalía Congénita
	Malformaciones
	Deformidades
	Defectos Congénitos
**Asfixia y trauma en el nacimiento**	
	Asfixia prenatal (descriptor)
**Cáncer de colon y recto**	
	Neoplasias del Colon (descriptor)
	Cáncer del Colon
	Cáncer de Colon

**Causa de defunción (palabra clave utilizada por OMS)**	**Sinónimos (DeCS)**
	Tumores del Colon
	Neoplasias del Recto (descriptor)
	Cáncer de Recto
	Cáncer Rectal
	Tumores Rectales
**Cáncer de estómago**	
	Neoplasias Gástricas (descriptor)
	Cáncer de Estómago
	Cáncer Gástrico
	Neoplasias del Estómago
	Cáncer del Estómago
**Cáncer de mama**	
	Neoplasias de la Mama (descriptor)
	Cáncer de Mama
	Cáncer de la Mama
	Cáncer de Seno
	Cáncer Mamario
	Neoplasias Mamarias
	Tumores de Mama
	Tumores de la Mama
	Tumor de Seno
	Tumores de Seno
	Tumores Mamarios
**Cáncer de próstata**	
	Neoplasias de la Próstata (Descriptor)
	Cáncer de Próstata
	Cáncer de la Próstata
	Cáncer Prostático
	Neoplasias Prostáticas
	Tumores de la Próstata
	Tumores Prostáticos
**Cáncer de pulmón**	
	Neoplasias Pulmonares (descriptor)
	Neoplasia Pulmonar

**Causa de defunción (palabra clave utilizada por OMS)**	**Sinónimos (DeCS)**
	Neoplasias del Pulmón
	Cáncer del Pulmón
	Cáncer Pulmonar
**Cardiopatía isquémica**	
	Isquemia Miocárdica (descriptor)
**Cirrosis hepatica**	
	Cirrosis del Hígado
	Fibrosis Hepática
	Fibrosis del Hígado
**Complicaciones del parto prematuro**	
	Complicaciones del Trabajo de Parto (descriptor)
	Complicaciones del Parto
**Diabetes**	
	Diabetes Mellitus (descriptor)
**Enfermedad cardiaca hipertensiva**	
	Cardiopatías (descriptor)
	Enfermedades del Corazón
	Enfermedades Cardíacas
**Enfermedad de alzhéimer**	
	Demencia Senil
	Demencia Senil Aguda Confusa
	Demencia Tipo Alzheimer
	Demencia Presenil de Alzheimer
	Demencia Senil Tipo Alzheimer
**Enfermedad renal**	
	Enfermedades Renales (descriptor)
	Nefropatías
**Enfermedades diarréicas**	
	Disentería (descriptor)
	Diarrea Aguda
	Diarrea Infecciosa
	Diarrea Disentérica
	Enfermedad Diarreica Infecciosa
**Infección aguda de las vías respiratorias inferiores**	

Causa de defunción (palabra clave utilizada por OMS)	Sinónimos (DeCS)
	Infecciones del Sistema Respiratorio (descriptor)
	Infecciones de las Vías Respiratorias
	Infecciones del Aparato Respiratorio
	Infecciones del Tracto Respiratorio
	Infecciones Respiratorias
**Lesiones en carretera**	
	Accidentes de Tránsito (descriptor)
	Accidente de Tránsito
	Accidente de Tráfico
	Accidentes de Tráfico
	Accidentes de Transporte
**Malnutrición protéico-energética**	
	Desnutrición (descriptor)
	Malnutrición
	Subnutrición
	Subalimentación
**Neumopatía obstructiva crónica**	
	Enfermedad Pulmonar Obstructiva Crónica (descriptor)
	COAD
	Obstrucción Crónica del Flujo Aéreo
	Obstrucción del Flujo Aéreo Crónica
	Enfermedad Obstructiva Crónica de las Vías Aéreas
	Enfermedad del Pulmón Crónica Obstructiva
	Enfermedad Pulmonar Crónica Obstructiva
	EVOC
	EPOC
	Neumopatía Obstructiva Crónica
**Tuberculosis**	
	TB
**VIH/SIDA**	
	Síndrome de Inmunodeficiencia Adquirida (descriptor)

Causa de defunción (palabra clave utilizada por OMS)	Sinónimos (DeCS)
	SIDA
	Síndrome de Deficiencia Inmunológica Adquirida
	Síndrome de la Inmunodeficiencia Adquirida
**Violencia interpersonal**	
	Violencia (descriptor)
	Atrocidades
	Conducta de Ataque
	Violencia Social
