# Supplementary material for: Social media in public health: an analysis of national health authorities and leading causes of death in Spanish-speaking Latin American and Caribbean countries
Source: BMC Med Inform Decis Mak. 2017 Feb 3;17:16. doi: 10.1186/s12911-017-0411-y (PMC5291998; doi:10.1186/s12911-017-0411-y)
Supplement: Additional file 2: — Summary table of information about the presence of national health authorities on social media. (PDF 220 kb) [file 12911_2017_411_MOESM2_ESM.pdf]

Additional file 2. Presence of national health authorities on social media.

Country	Ministry	Facebook	Twitter	Youtube	Google+	Flickr	Instagram
ARGENTINA	Ministerio de Salud	http://www.facebook.com/msalnacion	http://twitter.com/msalnacion	http://www.youtube.com/msalnacion	https://plus.google.com/113411333997715470916/posts		
BOLIVIA	Ministerio de Salud y Deportes	https://www.facebook.com/minsaludbolivia	https://twitter.com/minsaludbolivia	http://www.youtube.com/user/MinSaludBolivia			
CHILE	Ministerio de Salud	https://www.facebook.com/pages/Ministerio-de-Salud-Chile/273168169362020?ref=ts	http://twitter.com/ministeriosalud	http://www.youtube.com/user/ministeriosaludchile		http://www.flickr.com/photos/minsal/	
COLOMBIA	Ministerio de Salud y protección social	https://www.facebook.com/MinSaludCol	https://twitter.com/MinSaludCol	http://www.youtube.com/user/MinSaludColPrensa	https://plus.google.com/+MinSaludColPrensa/posts		https://instagram.com/minsaludcol/
COSTA RICA	Ministerio de salud	http://www.facebook.com/pages/Ministerio-de-Salud-de-Costa-Rica/486567330136?fref=ts	https://twitter.com/msaludcr				
CUBA	Infomed. Portal de						

Additional file 2. Presence of national health authorities on social media.

Country	Ministry	Facebook	Twitter	Youtube	Google+	Flickr	Instagram
	Salud de Cuba						
DOMINICAN REP.	Ministerio de Salud Pública	https://www.facebook.com/SaludPublicaRD	https://twitter.com/saludpublicard	https://www.youtube.com/user/SaludPublicaRD			
ECUADOR	Ministerio de Salud Pública	https://www.facebook.com/SaludEcuador	https://twitter.com/Salud_Ec	http://www.youtube.com/user/EcuadorSalud		http://www.flickr.com/photos/saludecuador	
EL SALVADOR	Ministerio de Salud	http://www.facebook.com/salud.sv	http://twitter.com/minsalud	http://www.youtube.com/comunicacionesminsal			
GUATEMALA	Ministerio de Salud Pública y Asistencia Social	https://www.facebook.com/MinisteriodeSaludPublicayAsistenciaSocial?pnref=story	https://twitter.com/saludguatemala	http://www.youtube.com/user/webmasterSigsaMspas?feature=sub_widget_1	https://plus.google.com/114942600761182309447/posts		
HONDURAS	Secretaría de Salud	https://www.facebook.com/saludhn/info?tab=page_info	https://twitter.com/saludhn				
MEXICO	Secretaría de Salud	https://www.facebook.com/SecretariadeSaludMX	https://twitter.com/SSalud_mx	http://www.youtube.com/user/ssaludmex			
NICARAGUA	Ministerio de Salud						

Additional file 2. Presence of national health authorities on social media.

Country	Ministry	Facebook	Twitter	Youtube	Google+	Flickr	Instagram
PANAMA	Ministerio de Salud	https://www.facebook.com/minsapanama	https://twitter.com/minsa_panama	http://www.youtube.com/user/MINSAPMA			
PARAGUAY	Ministerio de Salud Pública y Bienestar Social	http://www.facebook.com/ministeriodesaludparaguay?fref=ts	http://www.mspbs.gov.py/v3/wp-content/themes/graphene/images/social/twitter.png	http://www.youtube.com/user/comunicacionensalud?feature=watch			
PERU	Ministerio de Salud	http://www.facebook.com/minsaperu?v=wall	http://twitter.com/#!/Minsa_Peru	http://www.youtube.com/user/minsaperu1#p/u		https://www.flickr.com/photos/minsaperu/	
URUGUAY	Ministerio de Salud Pública	https://www.facebook.com/MSPUruguay?rf=284193221592796	https://twitter.com/mspuruguay				
VENEZUELA	Ministerio del Poder Popular para la Salud		https://twitter.com/mppsalud_vzla				
