# Supplementary material for: Social media in public health: an analysis of national health authorities and leading causes of death in Spanish-speaking Latin American and Caribbean countries
Source: BMC Med Inform Decis Mak. 2017 Feb 3;17:16. doi: 10.1186/s12911-017-0411-y (PMC5291998; doi:10.1186/s12911-017-0411-y)
Supplement: Additional file 3: — Summary table of the analysis of the use of Facebook, Twitter and YouTube by national health authorities. (PDF 392 kb) [file 12911_2017_411_MOESM3_ESM.pdf]

Additional file 3. Analysis of the use of Facebook, Twitter and YouTube by national health authorities

Facebook

Country	Nbr. followers	Posts per day	Users' post per day	Comments in fans' posts	Fans' posts	"Shares" per post	"Comments" per post	"Likes" per post	Number of "Shares"	Number of "Comments"	Number of "Likes"	Number of posts	Total of "Likes", "Comments", "Shares"	Growth (total)	Daily (%)	Percentage of increase from time of start	Total of users' posts, total of "Likes", "Comments" and "Shares"	Videos (time-sec.)
Argentina	221511	1,781,420,765	0	0	0	3,483,404,908	1,757,055,215	5,166,717,791	113559	5728	168435	326	287722	25154	12,79%	12,79%	0	0
Bolivia	7400	5,2	0	0	0	1,7	0,4	11	1600	379	10000	956	12000	122	1,7%	1,7%	0	254
Chile	71073	7,710,382,514	0	0	0	2,871,367,824	291,070,163	7,345,641,389	40515	4107	103647	1411	148269	5042	7,62%	7,62%	0	0
Colombia	36257	1,617,486,339	1,519,125,683	26	278	6,491,891,892	7,381,756,757	1,371,655,405	19216	2185	40601	296	62002	3191	9,65%	9,65%	94	103
Costa Rica	70355	92,896,175	1,535,519,126	79	281	5,305,882,353	5	6,341,176,471	902	85	1078	17	2065	1795	2,62%	2,62%	362	219
Ecuador	72975	131,147,541	1,071,038,251	30	196	407,625	7,083,333,333	9,727,083,333	9783	1700	23345	240	34828	22540	44,69%	44,69%	126	0

Additional file 3. Analysis of the use of Facebook, Twitter and YouTube by national health authorities

Country	Nbr. followers	Posts per day	Users' post per day	Comments in fans' posts	Fans' posts	"Shares" per post	"Comments" per post	"Likes" per post	Number of "Shares"	Number of "Comments"	Number of "Likes"	Number of posts	Total of "Likes", "Comments", "Shares"	Growth (total)	Daily (%)	Percentage of increase from time of start	Total of users' posts, total of "Likes", "Comments" and "Shares"	Videos (time-sec.)
El Salvador	11481	1,666,666,667	530,054,645	28	97	7,016,393,443	108,852,459	2,456,393,443	2140	332	7492	305	9964	470	4,27%	4,27%	60	98
Guatemala	6016	1,595,628,415	0	0	0	1,107,876,712	77,739,726	1,637,328,767	3235	227	4781	292	8243	424	7,57%	7,57%	0	0
Honduras	1960	2,759,562,842	71,038,251	0	13	3,871,287,129	497,029,703	1,637,821,782	1955	251	8271	505	10477	124	6,75%	6,75%	0	31
México	158021	6,715,846,995	0	0	0	1,462,034,174	3,362,896,664	2,022,595,606	179684	4133	248577	1229	432394	49593	45,59%	45,59%	0	809
Panamá	24858	612,021,858	333,333,333	3	61	5,142,857,143	964,285,714	1,616,071,429	576	108	1810	112	2494	46	0,19%	0,19%	7	57
Paraguay	22823	1,021,311	1,191,256,831	56	218	6,685,928,304	455,323,703	2,029,320,492	12496	851	37928	1869	51275	385	1,72%	1,72%	108	0

Additional file 3. Analysis of the use of Facebook, Twitter and YouTube by national health authorities

Country	Nbr. followers	Posts per day	Users' post per day	Comments in fans' posts	Fans' posts	"Shares" per post	"Comments" per post	"Likes" per post	Number of "Shares"	Number of "Comments"	Number of "Likes"	Number of posts	Total of "Likes", "Comments", "Shares"	Growth (total)	Daily (%)	Percentage of increase from time of start	Total of users' posts, total of "Likes", "Comments" and "Shares"	Videos (time-sec.)
		,475																
Perú	137313	1,297,814,208	0	0	0	5,107,915,789	4,279,578,947	1,262,938,947	121313	10164	299948	2375	431425	22999	20,08%	20,08%	0	5331
Rep. Dominicana	11477	2,021,857,923	0	0	0	4,2	421,621,622	9,035,135,135	1554	156	3343	370	5053	337	3,02%	3,02%	0	0
Uruguay	2822	426,229,508	322,404,372	13	59	3,101,282,051	1,282,051,282	2,608,974,359	2419	100	2035	78	4554	172	6,49%	6,49%	60	326
	57000	3,8	0,4	16	80	54	3,6	90	34000	2000	64000	692	100000	8800	12%	12%	54	482

Source: Facebook.com / Fanpagekarma.com

Additional file 3. Analysis of the use of Facebook, Twitter and YouTube by national health authorities

Twitter

Country	Page	Tweets	Tweets-day	Favorites	Retweets	Conversations	Retweets and Favorites	Followers	Retweets and Favorites/tweet	Favorites/tweet	Retweets/tweet	Interactions/tweet
Argentina	Ministerio de Salud de la Nación Argentina	741	4.049180328	6429	10370	0.00%	16799	109608	13.99	8.67	5.32	0.02%
Bolivia	Ministerio de Salud y Deportes, Bolivia	908	4.961748634	1189	1098	0.44%	2287	4964	1.2	1.3	0.1	0.06%
Chile	Ministerio de Salud - Chile	930	5.081967213	2406	8734	3.66%	11140	106539	9.39	2.58	6.81	0.01%
Colombia	Ministerio de Salud y Protección Social Colombia	3060	16.72131148	8274	12830	0.23%	21104	172519	4.19	2.7	1.49	0.00%
Costa Rica	Ministerio de Salud de Costa Rica	32	0.174863388	14	21	3.13%	35	37462	0.65	0.43	0.22	0.00%
Dominican Rep.	Ministerio de Salud Pública Rep. Dominicana	856	4.677595628	601	2456	0.00%	3057	29285	2.86	0.7	2.16	0.01%
Ecuador	Ministerio Salud Ecuador	1863	10.18032787	4046	12342	18.25%	16388	162723	6.62	2.17	4.45	0.01%
El Salvador	Ministerio de Salud de El Salvador	1409	7.699453552	1411	2628	11.85%	4039	34165	1.86	1	0.86	0.01%
Guatemala	Ministerio de Salud Pública y Asistencia Social Guatemala	462	2.524590164	231	616	7.79%	847	4215	1.33	0.5	0.83	0.05%
Honduras	Secretaría de Salud Honduras 2014-2018	1005	5.491803279	3743	14278	0.50%	18021	3074	14.2	3.72	10.48	0.69%
Mexico	Secretaría de Salud México	2088	11.40983607	16447	39717	0.00%	56164	305762	19.02	7.87	11.15	0.01%

Additional file 3. Analysis of the use of Facebook, Twitter and YouTube by national health authorities

Country	Page	Tweets	Tweets-day	Favorites	Retweets	Conversations	Retweets and Favorites	Followers	Retweets and Favorites/tweet	Favorites/tweet	Retweets/tweet	Interactions/tweet
Panamá	MINSA Panama	832	4.546448087	2292	4164	0.72%	6456	25804	5	2.75	2.25	0.03%
Paraguay	Ministerio de Salud - Paraguay	755	4.12568306	941	1791	2.52%	2732	36000	2.37218543	1.246357616	1.13	0.01%
Peru	Ministerio de Salud del Perú	1866	10.19672131	4267	7179	0.96%	11446	205302	3.84	2.28	1.56	0.00%
Uruguay	Ministerio de Salud Pública - Uruguay	478	2.612021858	1529	1571	2.09%	3100	2384	3.28	3.19	0.09	0.36%
Venezuela	Ministero Salud - Venezuela	3510	19.18032787	1565	16894	0.48%	18459	23357	4.81	0.44	4.37	0.02%
	Mean values	1300	7.1	3400	8500	3.30%	12000	81000	5.9	2.6	3.3	0.08%

Source: Twitter.com / Fanpagekarma.com

Additional file 3. Analysis of the use of Facebook, Twitter and YouTube by national health authorities

Youtube

Page	Number of followers	Number of total views	Number of total videos	Number of videos	Number of total views	Number of views per video	Like	Don't like	Comments
ministeriosaludchile	2047	3865983	136	26	1079331	4,151,273,077	375	147	136
minsalud bolivia	110	38087	198	8	1247	155,875	8	0	0
Minsa Panama	57	15016	106	15	1100	7,333,333,333	13	0	2
Minsa Peru	1212	497487	750	135	44983	3,332,074,074	207	7	23
comunicacionensalud	96	37938	207	24	1445	6,020,833,333	17	0	1
SaludPublicaRD	110	27861	47	4	723	180,75	2	0	6
MinSaludCol	2031	3581313	432	72	686414	9,533,527,778	226	71	47
MsalNacion	2031	1115157	845	90	103814	1,153,488,889	473	26	50
ssaludmex	1012	2623881	380	8	8239	1,029,875	70	8	6
Ministerio de Salud El Salvador	186	81664	267	15	1198	7,986,666,667	5	0	0
Salud Ecuador	749	244662	248	26	8116	3,121,538,462	38	6	5
Ministerio de Salud Pública y Asistencia Social (MSPAS)	94	37308	138	13	1248	96	1	0	3
Mean values	811	1000000	313	36	161000	4500	120	22	23

Source: Youtube.com / Fanpagekarma.com
