# Supplementary material for: Social media in public health: an analysis of national health authorities and leading causes of death in Spanish-speaking Latin American and Caribbean countries
Source: BMC Med Inform Decis Mak. 2017 Feb 3;17:16. doi: 10.1186/s12911-017-0411-y (PMC5291998; doi:10.1186/s12911-017-0411-y)
Supplement: Additional file 4: — Summary table of information about the population with Internet access following national health authorities by country on Facebook, Twitter and YouTube. (PDF 132 kb) [file 12911_2017_411_MOESM4_ESM.pdf]

Additional file 4. Population with Internet access following national health authorities by country on Facebook, Twitter and Youtube.

Country	Population	Internet population within a country	Internet population within a country (%)	Facebook (users who follow national health authority)	Facebook Internet access vs Followers (%)	Twitter (users who follow national health authority)	Twitter Internet access vs Followers (%)	Youtube (users who follow national health authority)	Youtube Internet access vs Followers (%)
Argentina	42,980,026	27,808,077	64.70	221,511	0,79	109,608	0,39	2,031	0,0007
Bolivia	10,561,887	4,121,248	39.02	7,400	0,17	4,964	0,12	110	0,002
Chile	17,762,647	12,851,275	72.35	71,073	0,55	106,539	0,82	2,047	0,01
Colombia	47,791,393	25,123,935	52.57	36,257	0,14	172,519	0,68	2,031	0,008
Costa Rica	4,757,606	2,350,733	49.41	70,355	2,99	37,462	1,59	-	-
Cuba	11,379,111	3,413,733	30.00	-	-	-	-	-	-
Dominican Republic	10,405,943	5,159,267	49.58	11,477	0,22	29,285	0,56	110	0,002
Ecuador	15,902,916	6,838,254	43.00	72,975	1,06	162,723	2,37	749	0,01
El Salvador	6,107,706	1,813,989	29.70	11,481	0,63	34,165	1,88	186	0,01
Guatemala	16,015,494	3,747,626	23.40	6,016	0,16	4,215	0,11	94	0,002
Honduras	7,961,680	1,519,089	19.08	1,960	0,13	3,074	0,20	-	-
Mexico	125,385,833	55,658,771	44.39	158,021	0,28	305,762	0,54	1,012	0,001
Nicaragua	6,013,913	1,058,449	17.60	-	-	-	-	-	-
Panama	3,867,535	1,737,297	44.92	24,858	1,43	25,804	1,48	57	0,003
Paraguay	6,552,518	2,817,583	43.00	22,823	0,81	36,000	1,27	96	0,003
Peru	30,973,148	12,451,205	40.20	137,313	1,10	205,302	1,64	1,212	0,009
Uruguay	3,419,516	2,101,635	61.46	2,822	0,13	2,384	0,11	-	-
Venezuela	30,693,827	17,495,481	57.00	-	-	23,357	0,13	-	-
**Mean values**		**10,448,202**	**43.41**		**0,79**		**0,86**		**0,005**
Source: The World Bank Group (WB), International Telecommunication Union (ITU), Owloo.									
