# Supplementary material for: Social media in public health: an analysis of national health authorities and leading causes of death in Spanish-speaking Latin American and Caribbean countries
Source: BMC Med Inform Decis Mak. 2017 Feb 3;17:16. doi: 10.1186/s12911-017-0411-y (PMC5291998; doi:10.1186/s12911-017-0411-y)
Supplement: Additional file 5: — Summary table showing a comparison of activity rate and information retrieval on Facebook and Twitter is included. (PDF 321 kb) [file 12911_2017_411_MOESM5_ESM.pdf]

**Additional file 5. Comparison of activity rate and information retrieval on Facebook and Twitter**

| Country | Activity Rate on Facebook | Number of leading causes of death found on Facebook | Leading causes of death found on Facebook | Activity Rate on Twitter | Number of leading causes of death found on Twitter | Leading causes of death found on Twitter |
|---|---|---|---|---|---|---|
| Argentina | 1.29 | 1 | - Hypertensive heart disease | 0.15 | 1 | - Stroke |
| Bolivia | 1.62 | 2 | - Diabetes<br>- Acute lower respiratory infection | 0.46 | 3 | - Stroke<br>- Diabetes<br>- Alzheimer's disease |
| Chile | 2.08 | 4 | - Stroke<br>- Stomach cancer<br>- Diabetes<br>- Alzheimer's disease | 0.1 | 4 | - Stroke<br>- Stomach cancer<br>- Diabetes<br>- Alzheimer's disease |
| Colombia | 1.71 | 4 | - Stroke<br>- Congenital anomalies<br>- Diabetes<br>- Acute lower respiratory infection | 0.12 | 3 | - Diabetes<br>- Traffic accidents<br>- Interpersonal violence |
| Costa Rica | 0.02 | 0 | | 0.00093 | 0 | |
| Cuba | - | - | | - | - | |
| Dominican Republic | 0.44 | 5 | - Stroke<br>- Diabetes<br>- Hypertensive heart disease<br>- Traffic accidents<br>- HIV/AIDS | 0.1 | 3 | - Stroke<br>- Diabetes<br>- Traffic accidents |
| Ecuador | 0.47 | 2 | - Diabetes<br>- HIV/AIDS | 0.1 | 2 | - Stroke<br>- Diabetes |

**Additional file 5. Comparison of activity rate and information retrieval on Facebook and Twitter**

| Country | Activity Rate on Facebook | Number of leading causes of death found on Facebook | Leading causes of death found on Facebook | Activity Rate on Twitter | Number of leading causes of death found on Twitter | Leading causes of death found on Twitter |
|---|---|---|---|---|---|---|
| El Salvador | 0.86 | 5 | - Diabetes<br>- Kidney disease<br>- Traffic accidents<br>- HIV/AIDS<br>- Interpersonal violence | 0.11 | 7 | - Stroke<br>- Diabetes<br>- Kidney disease<br>- Acute lower respiratory infection<br>- Traffic accidents<br>- HIV/AIDS<br>- Interpersonal violence |
| Guatemala | 1.37 | 5 | - Stroke<br>- Diabetes<br>- Diarrheal disease<br>- HIV/AIDS<br>- Interpersonal violence | 0.2 | 4 | - Stroke<br>- Diabetes<br>- Diarrheal disease<br>- HIV/AIDS |
| Honduras | 5.34 | 2 | - HIV/AIDS<br>- Interpersonal violence | 5.86 | 3 | - Stroke<br>- HIV/AIDS<br>- Interpersonal violence |
| Mexico | 2.73 | 4 | - Stroke<br>- Diabetes<br>- Traffic accidents<br>- Interpersonal violence | 0.18 | 3 | - Stroke<br>- Diabetes<br>- Interpersonal violence |
| Nicaragua | - | - | | - | - | |
| Panama | 0.10 | 1 | - HIV/AIDS | 0.25 | 1 | - HIV/AIDS |

**Additional file 5. Comparison of activity rate and information retrieval on Facebook and Twitter**

| Country | Activity Rate on Facebook | Number of leading causes of death found on Facebook | Leading causes of death found on Facebook | Activity Rate on Twitter | Number of leading causes of death found on Twitter | Leading causes of death found on Twitter |
|---|---|---|---|---|---|---|
| Paraguay | 2.24 | 7 | - Congenital anomalies<br>- Complications from premature birth<br>- Diabetes<br>- Hypertensive heart disease<br>- Acute lower respiratory infection<br>- Traffic accidents<br>- Interpersonal violence | 0.07 | 4 | - Stroke<br>- Diabetes<br>- Hypertensive heart disease<br>- Interpersonal violence |
| Peru | 3.14 | 6 | - Stroke<br>- Congenital anomalies<br>- Stomach cancer<br>- Diabetes<br>- Acute lower respiratory infection<br>- Traffic accidents | 0.05 | 3 | - Stroke<br>- Diabetes<br>- Traffic accidents |
| Uruguay | 1.61 | 0 | | 1.3 | 3 | - Stroke<br>- Alzheimer’s disease<br>- Acute lower respiratory infection |
| Venezuela | 1.29 | - | | 0.79 | 3 | - Stroke<br>- Diabetes<br>- Acute lower respiratory infection |
| **Mean values** | **1.66** | **3.2** | | **0.61** | **2.93** | |
